# Atypical White Matter Connectivity in Dyslexic Readers of a Fairly Transparent Orthography

**DOI:** 10.3389/fpsyg.2018.01147

**Published:** 2018-07-10

**Authors:** Gojko Žarić, Inge Timmers, Patty Gerretsen, Gorka Fraga González, Jurgen Tijms, Maurits W. van der Molen, Leo Blomert, Milene Bonte

**Affiliations:** ^1^Department of Cognitive Neuroscience, Faculty of Psychology and Neuroscience, Maastricht University, Maastricht, Netherlands; ^2^Maastricht Brain Imaging Center (M-BIC), Maastricht, Netherlands; ^3^Department of Anesthesiology, Perioperative, and Pain Medicine, Stanford University School of Medicine, Stanford, CA, United States; ^4^Regionaal Instituut voor Dyslexie, Maastricht, Netherlands; ^5^Department of Developmental Psychology, University of Amsterdam, Amsterdam, Netherlands; ^6^IWAL Instituut Voor Leerproblemen, Amsterdam, Netherlands

**Keywords:** developmental dyslexia, structural connectivity, diffusion tensor imaging, reading network, anterior thalamic radiation, arcuate fasciculus

## Abstract

Atypical structural properties of the brain’s white matter bundles have been associated with failing reading acquisition in developmental dyslexia. Because these white matter properties may show dynamic changes with age and orthographic depth, we examined fractional anisotropy (FA) along 16 white matter tracts in 8- to 11-year-old dyslexic (DR) and typically reading (TR) children learning to read in a fairly transparent orthography (Dutch). Our results showed higher FA values in the bilateral anterior thalamic radiations of DRs and FA values of the left thalamic radiation scaled with behavioral reading-related scores. Furthermore, DRs tended to have atypical FA values in the bilateral arcuate fasciculi. Children’s age additionally predicted FA values along the tracts. Together, our findings suggest differential contributions of cortical and thalamo-cortical pathways to the developing reading network in dyslexic and typical readers, possibly indicating prolonged letter-by-letter reading or increased attentional and/or working memory demands in dyslexic children during reading.

## Introduction

Reading is a complex cognitive function, requiring matching of visual symbols both to the sound units of speech and directly to word meaning (Ehri, 2005; Nation, 2009; Blomert, 2011). Nonetheless, it is mastered by 90–95% of children without notable problems. Conversely, children suffering from developmental dyslexia, a specific reading disability with neurobiological origin and a genetic component (Shaywitz and Shaywitz, 2005), fail to achieve proficient reading skills despite adequate cognitive abilities and educational opportunities (Lyon et al., 2003; Blomert, 2005). A lack of reading fluency has been pointed out as the most severe impairment (Shaywitz et al., 2008), particularly in relatively transparent orthographies such as Dutch and German (Wimmer and Schurz, 2010). Findings of abnormal functional and anatomical (white matter) connectivity within the brain’s reading network in dyslexia have been taken to suggest an underlying problem in information transfer within this network (Geschwind, 1965; Paulesu et al., 1996; Klingberg et al., 2000; Wimmer and Schurz, 2010). So far, most evidence for white matter abnormalities in dyslexia comes from studies comparing groups of dyslexic and typical readers of English. Given the dynamic and idiosyncratic nature of reading and white matter development (e.g., Yeatman et al., 2012a), it is essential to extend these findings to children at different developmental stages as well as to orthographies with various levels of orthographic-phonological transparency (e.g., Holloway et al., 2015). Here we investigate patterns of white matter connectivity in a group of 8- to 11-year-old dyslexic and TR children after receiving 2–3 years of reading instruction in a moderately transparent orthography (Dutch).

As a phylogenetically recent cognitive function, reading has been proposed to build on dominantly left lateralized neuronal networks for visual object recognition (Schlaggar and McCandliss, 2007) and spoken language (Liberman et al., 1974; Pugh et al., 2001b). These networks are located along the posterior to anterior axis of the brain and include ventral occipito-temporal, posterior-temporal, dorsal parieto-temporal, and inferior-frontal regions. These areas have each been associated with specific cognitive functions important for reading. Hence, posterior-temporal and parieto-temporal areas are recruited during tasks requiring phonological processing (Pugh et al., 2001a) and multisensory grapheme-phoneme integration (Simos et al., 2002; van Atteveldt et al., 2004; Blomert, 2011). The ventral occipito-temporal region is involved in fast visual word recognition (Jobard et al., 2003; McCandliss et al., 2003), and the left inferior-frontal regions partake in speech production and phonological recoding of words (Sandak et al., 2004). As compared to the extensively studied cortical reading network, less is known about the involvement of subcortical regions, although regions central to attentional and gating functions such as the thalamus are obviously involved in reading- and language-related tasks (Hynd and Semrud-Clikeman, 1989; Crosson, 1999; Theofanopoulou and Boeckx, 2015). Comparison of dyslexic and TR has indicated abnormalities in functional activation (e.g., McCandliss and Noble, 2003; Hoeft et al., 2007; Richlan et al., 2009; Blomert, 2011) and connectivity (e.g., Wolf et al., 2010; van der Mark et al., 2011; Finn et al., 2014; Schurz et al., 2014), at multiple levels of the reading network. For example, during visual word rhyme judgment, dyslexic, relative to TR, showed reduced activation in left parietal and bilateral fusiform cortices, while they exhibited stronger activation in left inferior and middle frontal gyri, caudate, and thalamus (Hoeft et al., 2007). Furthermore, dyslexic, relative to TR, exhibited altered functional connectivity, with, e.g., reduced connectivity along the visual pathway, together with increased connectivity to limbic regions and persistent connectivity to a left-hemisphere anterior language region (Finn et al., 2014).

Next to functional measures of brain activity and connectivity, anatomical white matter connectivity provides important additional information about the organization of the brain’s reading network. Anatomical white matter connectivity can be measured noninvasively with diffusion-weighted imaging (DWI) and tractography (Beaulieu, 2002). DWI measures diffusion of water in the brain. Diffusion refers to the movement trajectories of water molecules as a result of random motion (Beaulieu, 2002). Diffusion can be isotropic, i.e., there is no preferred direction of diffusion, or anisotropic, i.e., along a preferred direction. Within the brain’s neural tissue, there are multiple constraints that affect diffusion of both extracellular and intracellular water molecules, such as cell membranes, cytoskeleton, and macromolecules (Johansen-Berg and Behrens, 2009). In gray matter, which does not have an oriented fiber structure, diffusivity is largely isotropic. On the contrary, white matter consists of large bundles of axons that are oriented in a coherent and parallel manner. The diffusion here is anisotropic as it is constrained by myelination and several other properties (e.g., axonal membranes and neurofibrils), and hence it is faster along the axons than perpendicular to them (Beaulieu, 2002). The usually reported index of anisotropy is fractional anisotropy (FA, which tells us whether the diffusion in a certain direction is faster than in other directions, i.e., whether diffusion can be described with an ellipsoid rather than a sphere. FA values have traditionally been used as an index of white matter integrity that scales with the amount of myelination, as disease-related demyelination leads to significant reductions in FA values (Assaf and Pasternak, 2008). More recently, it has been suggested that major determinants of diffusion can be divided into macroscopic geometric factors, such as fiber direction coherence, and microscopic factors, such as axon properties (e.g., axon caliber), while the amount of myelination has a more modulatory role (Vandermosten et al., 2012b).

Possible functional roles of white matter connectivity have been suggested in studies relating values of diffusivity measures to offline behavioral measures of language and reading skills. Studies in typically developing children found a positive relation between FA of the posterior limb of the left internal capsule and word identification scores (Beaulieu et al., 2005), between FA in the left temporal lobe and word reading (Nagy et al., 2004), the left inferior fronto-occipital fasciculus (IFOF) and orthographic knowledge/lexical reading (Vanderauwera et al., 2018), as well as between FA of callosal fibers that connect the temporal lobes and phonological awareness (Dougherty et al., 2007). A tract that has been often related to reading and reading-related skills is the left arcuate fasciculus (AF), a curved tract connecting the posterior superior temporal gyrus/sulcus (i.e., Wernicke’s area) to the inferior frontal gyrus (i.e., Broca’s area) (Catani et al., 2005). In particular, this tract has been suggested to mediate merging of speech sound sequences (letter sequences, word sequences; Wernicke’s area) with word meaning (Broca’s area), potentially subserving the acquisition of new languages and reading (Lopez-Barroso et al., 2013). In typical readers, FA values of the left AF have been found to be related to both functional activity of the posterior superior temporal sulcus (pSTS) during cross-modal rhyme judgments and behavioral response accuracy, independently of the age of the participants (Gullick and Booth, 2014). In another study with typical readers, volume changes in the left AF and superior corona radiata predicted reading outcome (Myers et al., 2014), while FA changes of the left AF, posterior corona radiata, and IFOF were associated with self- or parent-reported reading habit (Takeuchi et al., 2016). Moreover, an increase in FA of the temporo-parietal portion of the left AF has also been related to literacy acquisition in adults (Thiebaut De Schotten et al., 2014). Interestingly, a relation between dyslexia-related genes, white matter volume of the left temporo-parietal region linking the middle temporal gyrus with the inferior parietal lobe, and reading scores was found in Swedish speaking TR children and young adults (Darki et al., 2012). Similarly, a study in German speaking non-dyslexic children found a cluster in the left AF with different FA values dependent on the presence or absence of a dyslexia-related gene (Skeide et al., 2015). Moreover, FA values of this cluster were related to individual differences in children’s phonological awareness skills.

Studies comparing white matter connectivity in typical and impaired readers have been conducted mostly with English speaking adults, but also in Swedish, Brazilian Portuguese, Italian, German, and Flemish/Dutch adults. The findings of these studies include abnormal FA values in dyslexic readers in parieto-temporal regions, especially along the AF, but also along the superior longitudinal fasciculus (SLF; Richards et al., 2008; Steinbrink et al., 2008; Frye et al., 2011; Vandermosten et al., 2012a, 2013), the anterior and posterior limb of the left internal capsule and the corona radiata, connecting the thalamus to the cerebral cortex (Klingberg et al., 2000; Richards et al., 2008). Other group differences have been reported in FA values of the corpus callosum, including the body, splenium, and isthmus (Frye et al., 2008; Richards et al., 2008; Welcome and Joanisse, 2014), the left inferior longitudinal fasciculus (ILF; connecting the ventral occipital to the anterior temporal lobe) and the IFOF (connecting the ventral occipital lobe and the orbitofrontal cortex) (Rimrodt et al., 2010; Lebel et al., 2013; Marino et al., 2014). Most of these studies also report significant relations between diffusion weighted measures and diverse reading-related skills (but see Moreau et al., 2018a,b).

Similar group differences and relations to reading scores have been observed in studies comparing dyslexic and TR English children. Thus, group differences in FA values and their correlation with diverse reading-related skills have been found in both the left and the right AF and SLF (Deutsch et al., 2005; Carter et al., 2009; Rimrodt et al., 2010; Hoeft et al., 2011; Yeatman et al., 2011, 2012a; Saygin et al., 2013), the left internal capsule and the corona radiata (Niogi and McCandliss, 2006; Odegard et al., 2009), the corpus callosum (Odegard et al., 2009; Hasan et al., 2012), the left ILF and IFOF (Yeatman et al., 2012a), cerebellar-cortical (Fernandez et al., 2016), and thalamo-cortical connections (Fan et al., 2014). On the other hand, studies in relatively transparent orthographies, such as German or Dutch, are still scarce and include either pre-reading children at familial risk of dyslexia (Vandermosten et al., 2015) or poor spellers (Gebauer et al., 2012) rather than dyslexics. One study that included dyslexic German speaking children reported an association between multiple dyslexia-related genes and white matter volume of the left postcentral cortex, left cerebellum and cerebellar peduncles, and bilateral portions of the cerebellum (Skeide et al., 2016), while another study showed predominantly left-lateralized alteration of white matter in reading and spelling impaired children, albeit in a rather restricted sample, i.e., only nine children in the impaired group (Banfi et al., 2016). A recent study in Dutch speaking children that focused on bilateral AF and IFOF revealed differences between dyslexics and TR children in the bilateral AF and the left IFOF, but did not investigate other white matter tracts (Vanderauwera et al., 2017). It thus remains an open question whether dyslexic children who learn to read in more transparent orthographies will show the same pattern of white matter atypicality as those in the opaque English orthography. In particular, orthographic depth may influence reading strategies (Ziegler and Goswami, 2005), which may lead to subtle differences in the organization of the brain’s reading network (Martin et al., 2016), including a differential usage of the underlying white matter tracts and possibly a different relation of their FA values to reading (dys)fluency and skills.

The aim of the current study is to examine possible abnormalities along the white matter tracts of dyslexic readers learning to read in the fairly transparent Dutch orthography, thus expanding the previous findings from deeper orthographies and different age groups. To this end, we investigate structural white matter connectivity by analyzing DWI data in both TR (*n* = 13) and dyslexic children (*n* = 15). The sample consisted of 8- to 11-year-old children, as previous work of our group showed the strongest developmental changes in the neural reading network, and more specifically in the cross-modal integration of letters and speech sounds, in the age-period of 8 to 11 years: from hardly any signs of cross-modal integration after 1 year of formal reading education (∼8 years of age) to almost adult-like neural processing after 4 years of education (∼11 years of age) (Froyen et al., 2009; Blomert, 2011; Žarić et al., 2014; Fraga González et al., 2017). Our age-range represents a vital stage in the developmental path toward fluent reading, and from the perspective of dyslexia as a developmental disorder, thus zooms in at a stage where normal and abnormal pathways are expected to deviate. A subgroup of the dyslexic children in the current study exhibited a left lateralized functional deficit in letter-speech sound integration in a previous fMRI study (Blau et al., 2010). Because a similar audiovisual integration deficit has been shown to correlate with white matter connectivity along the AF in English readers (Gullick and Booth, 2014), we primarily expected differences in the left AF. However, given the divergence of DTI results reported in the literature, next to focusing on the left AF, we analyze a total of 16 white-matter tracts associated with dyslexia, reading, and the language network (e.g., Frye et al., 2008; Vandermosten et al., 2012b; Dick et al., 2013; Lebel et al., 2013; Fan et al., 2014; Nikki Arrington et al., 2017). In particular, these include the bilateral AF and SLF, ILF, IFOF, corpus callosum, thalamo-cortical connections, as well as corona radiata, the internal capsule through which the cortico-spinal tracts (CS) descend (e.g., Seo et al., 2012), and the uncinate fasciculus. We employ automatic fiber quantification (AFQ) to investigate group differences in the 16 white matter tracts, both in terms of their spatially averaged mean FA values and local FA values at hundred equally spaced nodes along each tract. Moreover, AFQ tracks fibers in subject’s native anatomical space, thus avoiding potential problems with tract alignment (Wassermann et al., 2011). Finally, to understand the relation between white matter and reading skills beyond group differences we correlate FA values and reading-related behavioral scores both across and within the groups of dyslexic and TR.

## Materials and Methods

### Participants

Participants were 44 native Dutch-speaking children, of whom 19 were typical readers (TR) and 25 were diagnosed as dyslexic readers (DR) (see below). All included dyslexic readers scored at least 1 standard deviation (SD) below the expected standard (age-appropriate) reading fluency and/or accuracy score. At the time of the study, the children had received between 1 and 4 years of reading instruction. All children had normal or corrected-to-normal vision and normal audition. Data of five TRs and five DRs were discarded from further analysis due to the excessive motion (see below for details), and one DR was discarded due to low DWI data quality for which the RESTORE algorithm could not be successfully performed. Because all of the remaining TR children went to Dutch study groups 5 and 6, i.e., they had received between 2 and 3 years of reading instruction, we further excluded five DR children that received either less (1 year; two DRs) or more (4 years; three DRs) than 2 to 3 years of reading instruction, thus leaving 15 dyslexic and 13 TR for the analysis (Age: *M*(SD) = 9.28(0.6) range: 8.08–11.25).

Typical readers were recruited via local schools. DRs were recruited from a national institute for dyslexia (RID), where they were diagnosed as dyslexic following an extensive cognitive psycho-diagnostic procedure. The two groups were matched for age and IQ (≥85, Dutch version of Wechsler Intelligence Scale for Children) and were tested on selected subtests (**Table 1**) of the 3DM (differential diagnostics for dyslexia) test battery (Blomert and Vaessen, 2009). The included subtests were a computerized reading test (high-frequency, low-frequency words, and pseudowords), a phoneme-deletion task, a spelling task, rapid naming, memory span of syllables, and a letter-speech sound discrimination task (Blomert and Vaessen, 2009). A score of overall reading accuracy (number of correctly read words/total number of read words) and fluency (number of correctly read words in 1.5 min) was derived from the 3DM reading test. Parents or caretakers signed written informed consent in accordance with the Declaration of Helsinki. The protocol was approved by the Ethical Review Committee Psychology and Neuroscience, Maastricht University.

**Table 1:** Behavioral reading scores: Descriptive data and statistical group comparisons for typical and dyslexic readers.

		Typical readers		Dyslexic readers				
*N*		13		15				
Sex ratio (m:f)		5:8		8:7				
Handedness (L:R)		1:12		2:13				
Years of reading instruction (2:3)		8:5		9:6				

		***M***	***SD***	***M***	***SD***	***F*(1,27)**	***P***	**$\eta_{\text{p}}^{2}$**

Age	(months)	111.69	8.44	111.13	6.29	0.40	0.843	0.002
Word reading	Accuracy (%)	98.80	1.76	90.53	5.35	28.27	<0.001^∗^	0.521
	Fluency (words/90 s)	118.46	24.59	65.60	20.21	38.99	<0.001^∗^	0.600
Word reading	Accuracy (T)^a^	58.37	4.69	35.74	12.71	36.73	<0.001^∗^	0.586
	Fluency (T)	52.99	9.65	30.84	7.55	46.37	<0.001^∗^	0.641
Spelling	Accuracy (%)	89.63	6.76	68.05	11.52	^Y^29.25	<0.001^∗^	0.549
	RT (s/item)	2.48	0.59	3.67	0.91	^Y^15.11	0.001^∗^	0.386
Letter-speech sound matching	Accuracy (%)	91.11	4.25	90.79	3.86	^X^0.04	0.848	0.002
	RT (s/item)	1.45	0.25	1.73	0.36	^X^4.87	0.038^∗^	0.175
Phoneme deletion	Accuracy (%)	84.58	14.07	64.59	15.24	^X^11.33	0.003^∗^	0.330
	RT (s/item)	2.49	0.85	5.55	2.15	^X^19.69	<0.001^∗^	0.461
Memory span (syllables)	Accuracy (%)	49.30	15.13	40.39	9.05	^X^3.36	0.080	0.127
Rapid naming – RAN (letters, digits, objects)	RT (s/stimulus card)	9.04	1.17	11.39	1.75	^W^14.32	<0.001^∗^	0.394
WISC	Similarities (SS)^b^	10.92	2.87	11.50	1.83	^Z^0.39	0.536	0.016
	Block design (SS)	9.92	2.81	10.57	2.28	^Y^0.44	0.515	0.017
	Digit-span (SS)	12.58	3.12	10.21	1.81	^Y^5.83	0.024^∗^	0.195

*^a^T scores (M = 50, SD = 10). ^b^Standard scores (M = 10, SD = 3). ^W^Df = (1,23), ^X^Df = (1,24), ^Y^Df = (1,25), ^Z^Df = (1,26), due to the missing data. ^∗^Denotes significant group differences.*

### DWI Data Acquisition

Diffusion weighted imaging (DWI) data was acquired using a Siemens 3T MAGNETOM Allegra MR scanner equipped with a high slew-rate head gradient-coil (amplitude 40 mT/m, slew rate 400 T/m/s) and an eight-channel phased-array head RF-coil. The DWI data were obtained using a doubly refocused spin echo EPI sequence. Due to a scanner update, two comparable protocols were used for DWI data acquisition. About half of the children from both groups that were included in the analysis were scanned with protocol one (TR: 8, DR: 8) and the other half with the protocol 2 (TR: 5, DR: 7). In the DWI protocols, 85 slices [voxel-size 1.8 mm isotropic; repetition time (TR) = 10800_(protocol1)_/11000_(protocol2)_ ms; echo time (TE) = 84_(protocol1)_/85_(protocol2)_ ms] were acquired at *b*-values of 1000 s/mm^2^, with 72 diffusion-encoding gradient directions. In addition, 3 (protocol 1) or 8 (protocol 2) *b* = 0 s/mm^2^ images were collected. Total acquisition time for the sequence was around 15 min. In addition to the DWI measurement, a high-resolution T1-weighted 3D MPRAGE scan (TR = 2250, TE = 2.6 ms, flip angle = 9°, 256 × 256 matrix, 192 sagittal slices, 1 × 1 × 1 mm voxels) was acquired for gray/white matter boundary segmentation. The child’s head was immobilized using foam cushions. Prior to the scanning session children performed a practice session in a “dummy” scanner to get used to the scanner environment and to practice to reduce movements.

### Diffusion Data Preprocessing

Pre-processing of the data was performed using mrDiffusion, a part of an open-source mrVista package from Stanford VISTA Lab^1^, and SPM8 working under MATLAB 2014a (The MathWorks, Natick, MA). Anterior and posterior commissures (AC and PC) were manually identified in the T1 weighted anatomical images of each subject and the anatomical images were rotated to the AC-PC plane. We performed brain extraction for T1-weighted images using the Robust Brain Extraction toolbox (Iglesias et al., 2011). The preprocessing of the diffusion data included estimation and correction of eddy current-induced distortions and subject motion by a non-linear co-registration (14 parameters) of an expected distortion based on the phase-encoding direction of the acquired data (Rohde et al., 2004) as implemented in mrDiffusion. Motion-corrected unweighted images were averaged and aligned to each individual child’s T1 weighted anatomical image. Diffusion-weighted images were individually registered to the obtained unweighted average by a two-stage coarse-to-fine approach, and eddy-current intensity correction was applied. The diffusion-weighting gradient directions were rotated using the resulting rotation component from the previous step. To ensure that FA differences were not the result of differences in head motion (Yendiki et al., 2014), we first performed visual inspection of motion correction indices and excluded volumes in which large spikes of movement occurred. Next, we calculated absolute displacement of each volume as root mean square values of the six motion parameters [three translations and three rotations, RMS_abs_ = √ [(x_n_^2^+y_n_^2^+z_n_^2^+α_n_^2^+β_n_^2^+γ_n_^2^)/N], where X_n_ denotes movement along the *x* axes relative to the first volume for volume *n* and so on (Theys et al., 2014). Conversion of rotational parameters from degrees to millimeters was calculated as a displacement on the surface of a sphere with a radius of 50 mm (Power et al., 2012; Theys et al., 2014). If the mean absolute displacement was larger than half of the voxel size, the subject was removed from the analysis (Power et al., 2012; Theys et al., 2014). In this step, we excluded data of five TR and five dyslexic children. Then, a robust least-squares algorithm was applied to fit the diffusion tensors and at the same time remove outliers from the tensor estimation step (Chang et al., 2005). One additional child from the dyslexic group was excluded as we could not run the RESTORE algorithm due to too much noise in the dataset. There were no differences in motion RMS_abs_ between the resulting two groups [*t*(27) = −0.143, *p* = 0.888]. The tensors were decomposed to the eigenvalues (λ_1_, λ_2_, λ_3_) that were used to compute FA (√(1/2)^∗^√((λ_1_ − λ_2_)^2^+(λ_2_ − λ_3_)^2^+(λ_3_ − λ_1_)^2^)/√(λ_1_^2^+λ_2_^2^+λ_3_^2^); Basser and Pierpaoli, 1996; Johnson et al., 2013).

### Fiber Tracking and Quantification

Fiber tracking and quantification was performed using the Automated Fiber Quantification package – AFQ (Yeatman et al., 2012b) under MATLAB. AFQ identifies 20 fiber tracts per subject using a three-step procedure combining deterministic tractography with a streamlines tracking algorithm (STT) (Mori et al., 1999; Basser et al., 2000), fiber tract segmentation based on waypoint regions of interest (Wakana et al., 2007) and fiber tract refinement based on a probabilistic fiber tract atlas (Hua et al., 2008).

For whole brain tractography, a white matter mask (all voxels with FA > 0.3) was used to seed a fourth-order Runge–Kutta path integration method with a step-size of 1-mm (8 seed points per voxel). The stopping criteria for streamlines were FA < 0.2 at the current position, a minimum angle between two consecutive segments greater than 30°, and a minimum fiber length of 20 mm (Verhoeven et al., 2010). In a next step, fibers were segmented as potentially belonging to one of 20 fiber groups if they passed through the regions defining the trajectory of the fascicle based on a white matter atlas (Wakana et al., 2007). These waypoint regions of interest (ROI) were positioned to extract the central portion of each tract, as it contains more coherent bundles of fibers than the end portions where fibers start to separate. Finally, fibers were compared to fiber tract probability maps (Hua et al., 2008) and discarded if they passed through regions of low probability.

**FIGURE 1:**
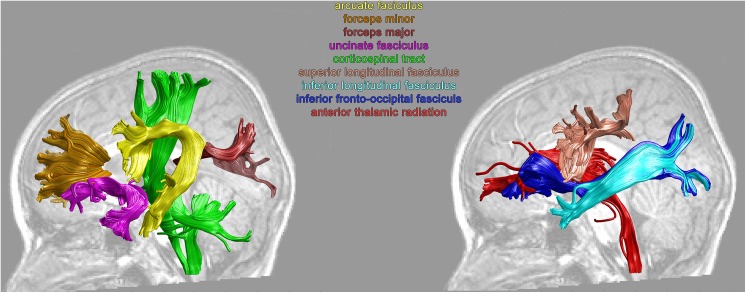
Examples of the individual left hemisphere white matter tracts from a TR child.

To control for possible errors in tractography (e.g., due to noise or complex fiber orientation), the fibers that were more than four standard deviations longer than the mean fiber length across subjects, or deviated more than five standard deviations from the core of the fiber tract were removed in an iterative process until there were no more outliers (for detailed description of the AFQ pipeline please see Yeatman et al., 2012b). Finally, we inspected resulting fibers and manually removed any remaining fibers that deviated from the expected anatomical pathways within the two ROIs (Yeatman et al., 2012b; Johnson et al., 2013; Wang et al., 2016). Overall, the shape and anatomical paths of the resulting white matter tracts (see **Figure 1** for an example) were comparable to these previous studies (Yeatman et al., 2012b; Johnson et al., 2013; Wang et al., 2016). Finally, fibers of each tract were resampled to 100 equal nodes, and FA was calculated at each node using spline interpolation of the diffusion properties. The FA values of the tract at each node were calculated as a weighted average of the diffusion properties at that node of each fiber (Yeatman et al., 2012b).

### Group Analysis

We analyzed 16 white-matter tracts associated with the brain’s reading and language network. Thus, tracts included in the analysis consisted of the bilateral arcuate fasciculi (AF), superior longitudinal fasciculi (SLF), ILF, IFOF, uncinate fasciculi (UF), CS, anterior-thalamic radiations (ThR), and forceps minor and forceps major (**Figure 1**). Due to the strict criterion for tract identification implemented in AFQ (Johnson et al., 2014), in one dyslexic subject the right IFOF was not identified, and the right AF was not tracked in one other dyslexic subject.

To evaluate group differences between TR and dyslexic children, we employed repeated measures analysis of covariance per tract (as implemented in MATLAB) with nodes as within subject factor (100 levels: 1–100), Group as between subject factor (2 levels: TR and dyslexic readers) and Age (in months) and Acquisition protocol (two levels) as covariates (for a similar approach see, e.g., Johnson et al., 2014). For completeness of results, we included Group^∗^Age and Group^∗^Protocol interactions in the model. As we performed repeated measures ANCOVAs for 16 tracts, we adjusted the significance threshold to 0.0031 (Bonferroni adjustment). Furthermore, as AFQ allows investigating FA changes along the tracts, in case of significant ANCOVA group effects we performed follow-up analysis, by employing unpaired *t*-tests for each node along the tract. To correct for multiple comparisons, we randomly shuffled participants’ data between groups (*n* = 10000; Nichols and Holmes, 2002) and estimated the minimum significant cluster of adjacent nodes for which typical readers had larger FA values than dyslexics (left tailed) and vice versa (right tailed). Thus, we limited our group analysis to clusters in which adjacent nodes exhibited group differences in the same direction, as this is more biologically plausible than opposite group differences in adjacent nodes. Furthermore, we calculated correlations across 100 nodes of each tract for which we obtained age and/or group effects in the rmANCOVA. We correlated FA values with age (in months) using simple correlation and with reading-related scores using partial correlations, controlling for age, group, and digit span (to account for group differences on this measure, see **Table 1**) when combining the two groups, and controlling for age when calculating correlations for each group separately. To account for the multiple comparisons for correlations obtained across 100 nodes, we used false discovery rate (FDR) correction (Benjamini and Hochberg, 1995) per tract per behavioral measure.

## Results

We first analyzed group differences using repeated measures ANCOVA with Nodes as within subject factor (100 levels: 1–100), Group as between subject factor (two levels: TR and dyslexic readers) and Age (in years), and Acquisition Protocol (two levels) as covariates. We used Bonferroni correction for multiple comparisons adjusting the significance level to *p* = 0.003.

**FIGURE 2:**
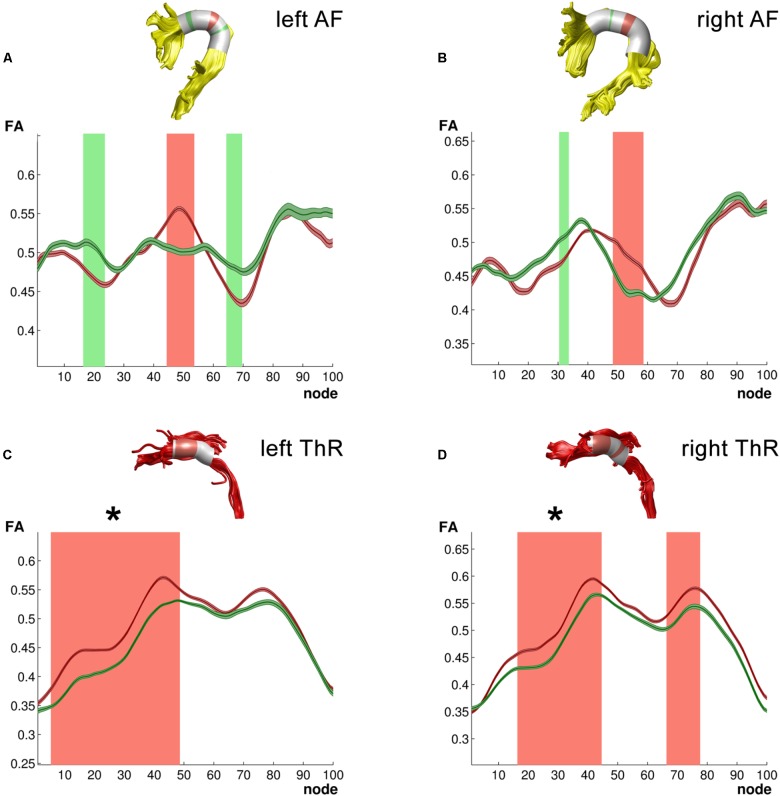
Tracts for which ANCOVA yielded group differences. **(A)** Left arcuate fasciculus (AF); **(B)** Right arcuate fasciculus (AF); **(C)** Left anterior thalamic radiation (ThR); **(D)** Right anterior thalamic radiation (ThR). All 3D reconstructions are examples from a single subject. Green – typical readers mean FA ± 1SE; Red – dyslexic readers mean FA ± 1SE; light green – higher FA values in typical readers at uncorrected level in a follow-up analysis; light red – higher FA values in dyslexic readers at uncorrected level in a follow-up analysis. Clusters of group differences that survived multiple comparison correction in the follow-up analysis are marked with asterisk (^∗^).

We first focused on the long association white matter tracts within the dorsal reading network including the bilateral arcuate (AF) and SLF. This analysis yielded significant group (Group^∗^Nodes: IAF: *F*_99,2178_ = 3.86, *p* < 0.003, $\eta_{\text{p}}^{2}$ = 0.15; rAF: *F*_99,2079_ = 1.59, *p* < 0.003, $\eta_{\text{p}}^{2}$ = 0.07) and age (Age^∗^Nodes interaction – IAF: *F*_99,2178_ = 5.07, *p* < 0.003, $\eta_{\text{p}}^{2}$ = 0.19; rAF: *F*_99,2079_ = 1.63, *p* < 0.003, $\eta_{\text{p}}^{2}$ = 0.07) differences in FA values across nodes in the left and right AF. Furthermore, in the left AF, group differences in these mean FA values were modulated by age (Group^∗^Age^∗^Nodes: *F*_99,2178_ = 3.38, *p* < 0.003, $\eta_{\text{p}}^{2}$ = 0.13). A follow-up analysis of group differences in FA values along 100 individual nodes of the left and right AF revealed three and two clusters, respectively, that were not large enough to reach the corrected significance threshold (**Figures 2A,B**). A further analysis correlating age with FA values along the 100 nodes showed no significant correlations for the left or right AF. Analysis of FA values across the SLF, yielded no significant effects for the left SLF, while the right SLF showed a significant effect of group across nodes (Group^∗^Node: *F*_99,2178_ = 3.32, *p* < 0.003, $\eta_{\text{p}}^{2}$ = 0.13). Furthermore, the right SLF yielded a significant effect of age across nodes (Age^∗^Nodes: *F*_99,2178_ = 2.57, *p* < 0.003, $\eta_{\text{p}}^{2}$ = 0.10), and this effect of age differed between the two groups (Group^∗^Age^∗^Nodes: *F*_99,2178_ = 2.95, *p* < 0.003, $\eta_{\text{p}}^{2}$ = 0.12). Follow-up analysis did not reveal significant group or age differences for individual clusters of nodes along the left or right SLF.

In the ventral reading pathways, we observed no group differences, but a significant age difference across the nodes of the left and right ILF (Age^∗^Nodes: lILF: *F*_99,2178_ = 1.80, *p* < 0.003, $\eta_{\text{p}}^{2}$ = 0.08; rILF: *F*_99,2178_ = 1.73, *p* < 0.003, $\eta_{\text{p}}^{2}$ = 0.07). Furthermore, for the right ILF the protocol played a significant role, i.e., there were significant Protocol^∗^Nodes (*F*_99,2178_ = 1.55, *p* < 0.003, $\eta_{\text{p}}^{2}$ = 0.07) and Group^∗^Protocol^∗^Nodes (*F*_99,2178_ = 1.45, *p* < 0.003, $\eta_{\text{p}}^{2}$ = 0.06) interactions. Finally, the other reading-related cortical tracts, i.e., the left and right UF and left and right IFOFs, showed no significant effects.

Considering commissural fibers, age affected FA values in both groups, with significant Age^∗^Nodes interaction across both the Callosum Forceps Major (*F*_99,2178_ = 2.36, *p* < 0.003, $\eta_{\text{p}}^{2}$ = 0.10) and the Callosum Forceps Minor (*F*_99,2178_ = 1.59, *p* < 0.003, $\eta_{\text{p}}^{2}$ = 0.07). Furthermore, across the Callosum Forceps Major we observed Group^∗^Nodes (*F*_99,2178_ = 1.94, *p* < 0.003, $\eta_{\text{p}}^{2}$ = 0.08) as well as Group^∗^Age^∗^Nodes interactions (*F*_99,2178_ = 2.25, *p* < 0.003, $\eta_{\text{p}}^{2}$ = 0.09). However, because the acquisition protocol significantly affected FA values of the Callosum Forceps Major (Protocol^∗^Nodes: *F*_99,2178_ = 1.59, *p* < 0.003, $\eta_{\text{p}}^{2}$ = 0.07; Group^∗^Protocol^∗^Nodes: *F*_99,2178_ = 1.76, *p* < 0.003, $\eta_{\text{p}}^{2}$ = 0.07), we did not proceed with the follow up analysis for this tract.

**FIGURE 3:**
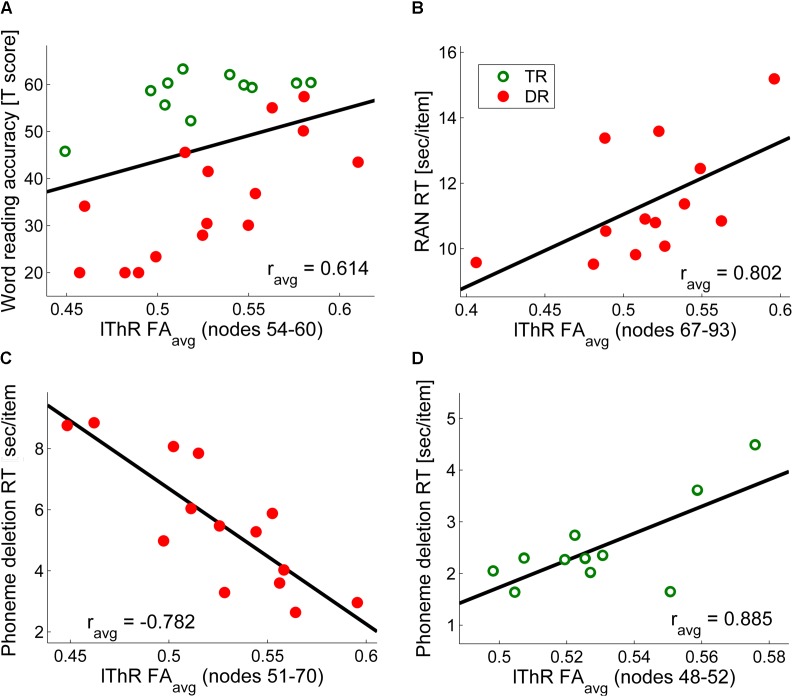
Correlations of FA values along the left anterior thalamic radiation with **(A)** word reading accuracy across the two groups (controlled for age, group and digit span) **(B)** phoneme deletion reaction time for dyslexic readers only (controlled for age) **(C)** rapid automatized naming reaction time for dyslexic readers only (controlled for age) **(D)** phoneme deletion reaction time for typical readers only (controlled for age). For visualization purposes we calculated mean values of significant correlations (e.g., in **(A)**, the significant correlations were observed along nodes 54–60, and the graph in **(A)** represents the average correlation value over these seven nodes).

Finally, dyslexic and TR differed in FA values across nodes of the bilateral anterior ThR (Group^∗^Nodes: lThR: *F*_99,2178_ = 3.11, *p* < 0.003, $\eta_{\text{p}}^{2}$ = 0.12; rThR: *F*_99,2178_ = 3.52, *p* < 0.003, $\eta_{\text{p}}^{2}$ = 0.14). Furthermore, both anterior ThR showed an age effect across nodes (Age^∗^Nodes: lThR: *F*_99,2178_ = 3.44, *p* < 0.003, $\eta_{\text{p}}^{2}$ = 0.14; rThR: *F*_99,2178_ = 3.82, *p* < 0.003, $\eta_{\text{p}}^{2}$ = 0.15), and these age effects differed between the two groups (Group^∗^Age^∗^Nodes: lThR: *F*_99,2178_ = 3.05, *p* < 0.003, $\eta_{\text{p}}^{2}$ = 0.12; rThR: *F*_99,2178_ = 3.36, *p* < 0.003, $\eta_{\text{p}}^{2}$ = 0.13). Follow-up analysis yielded clusters of significantly higher FA values in dyslexic relative to typical readers in the left anterior ThR (nodes 6–48, *p*_cluster_size_ = 0.002; **Figure 2C**) as well as in the right anterior ThR (nodes 17–44, *p*_cluster_size_ = 0.001; **Figure 2D**). Interestingly, FA values of the left anterior ThR were positively related to age appropriate reading accuracy scores across the two groups (nodes 54–60, *r_avg_* = 0.614, *p_fdr_avg_* = 0.002; controlled for age, group and digit span; **Figure 3A**). Furthermore, within the dyslexic group, FA values of the left ThR were negatively related to children’s reaction time on a phoneme deletion task (nodes 51–70, *r_avg_* = −0.782, *p_fdr_avg_* = 0.002; controlled for age; **Figure 3C**) and positively related to their reaction time for rapid automatized naming (nodes 67–93, *r_avg_* = 0.802, *p_fdr_avg_* = 0.003; controlled for age; **Figure 3B**). Conversely, within the group of typical readers, FA values of the left ThR were positively related to children’s reaction time on a phoneme deletion task (nodes 48–52, *r_avg_* = 0.885, *p_fdr_avg_* < 0.001; controlled for age; **Figure 3D**). No significant age or group effects were observed for the CS.

Given the apparent difference between groups with regard to digit span (i.e., lower scores for dyslexics) we performed a subsidiary analysis including digit span scores as a covariate, as suggested by a reviewer. The results that emerged from this analysis revealed that individual differences in digit span score did not influence the basic pattern of results. That is the observed differences in the bilateral ThRs, AFs, and right SLF were still present. This analysis, however, did influence the results for Corpus Callosum Major, for which interactions including Protocol were not significant anymore. The follow-up analysis did not reveal any significant clusters (at uncorrected level, FA values were higher in TRs in nodes 5–6, and 46–48).

## Discussion

In the present study, we examined structural white-matter connectivity in TR and dyslexic children after 2–3 years of reading instruction. To this end, we performed deterministic tractography and quantified both the mean FA values for 16 white matter tracts and the FA values across hundred equally spaced nodes along each tract. Our results indicate group differences primarily in the bilateral anterior ThR, as well as in language- and reading-related tracts, specifically in the bilateral arcuate (AF) and right superior longitudinal (SLF). Interestingly, children’s age was an important additional predictor of FA values in these tracts. Finally, reading and phonological processing skills significantly correlated with FA values along the left ThR, both across and within the two groups.

### Higher FA Values of the Bilateral Thalamic Radiations in Dyslexic Children Scale With Reading-Related Scores

Dyslexic children, relative to typical readers, exhibited higher FA values along the bilateral anterior ThR and the FA values of the left ThR scaled with reading-related behavioral scores across and within the two groups. Although the thalamus is not typically related to reading dysfunction and dyslexia, the anterior ThR passes through the anterior limb of the internal capsule and corona radiata, both implicated in reading (e.g., Beaulieu et al., 2005; Qiu et al., 2008) and dyslexia (e.g., Niogi and McCandliss, 2006; Richards et al., 2008). The anterior ThR connects the hypothalamus and limbic structures with the frontal cortex and anomalies along this tract have, for example, been related to deficiencies in working memory and executive function in schizophrenia (Mamah et al., 2010). A recent study found increased connectivity between the thalamus and both the lateral prefrontal cortex and the sensorimotor cortex in English-speaking dyslexic children (Fan et al., 2014). Furthermore, Fan et al. (2014) observed a negative correlation between thalamo-cortical connectivity and a composite score of visual word identification and word attack measures, while another study found a negative correlation between thalamo-cortical connections and pseudoword reading (Davis et al., 2010). Contrary to the increased anterior thalamo-cortical connectivity, more posterior thalamo-cortical connectivity, i.e., between LGN and V5/MT may be reduced in dyslexia (Müller-Axt et al., 2017). Interestingly, previous cytohistological studies have suggested abnormal cytoarchitecture in thalamic nuclei that relay auditory and visual, but also multimodal information in dyslexic readers (Galaburda and Eidelberg, 1982; Galaburda et al., 1985; Livingstone et al., 1991).

Increased thalamo-cortical connectivity toward sensory motor cortex has been hypothesized to represent “a prolonged multisensory engagement phase” in dyslexic children (Fan et al., 2014). In other words, the multisensory integration of letters and speech sounds, a first step in reading acquisition, may be prolonged and/or impaired in dyslexia (Blomert, 2011), leading to prolonged letter by letter reading in dyslexic children, instead of shifting to rapid visual word recognition (Share, 1995; Ehri, 2005). Such prolonged letter by letter reading and halted integration of orthography to meaning (Snowling, 1980) might explain the relation between left ThR FA values and reading-related behavioral scores in the current study. Thus, in the current study, higher FA values of the left ThR were related to more accurate word reading across the two groups, faster phoneme deletion in the dyslexic readers, and slower phoneme deletion in typical readers. Although opposite relations of FA and speed of phoneme deletion in dyslexic and TR may suggest that ThR subserves a compensatory mechanism in dyslexia, the question remains whether the observed FA differences are a consequence or cause of the reading difficulties. Interestingly, higher FA values of the left ThR were also related to slower reaction time on RAN in the group of dyslexic readers. Although this may seem as a contradictory result, it is important to note that although both RAN and phoneme deletion measure reading-related skills, these skills may be dissociable, which for example led some authors to argue for a double deficit in dyslexia based on the absence of deficiencies either in rapid naming and/or phonological awareness (Wolf and Bowers, 1999). Moreover, RAN and phonological awareness seem to affect reading fluency in school aged children, but while the relationship of phonological awareness with reading fluency decreases with reading experience, the influence of RAN increases over the years of reading instruction (Vaessen and Blomert, 2010). Thus, the children with 2 to 3 years of reading instruction included in our study, and especially the dyslexic children, may still largely rely on letter by letter reading. It would be important though to confirm this relation with reading level and/or strategies in future studies following dyslexic readers as well as average and excellent readers while their reading skills develop.

Alternatively, increased FA in the ThR could be a consequence of increased attentional (Crick, 1984; Cropley et al., 2006) or working memory (Crosson et al., 1999; Crottaz-Herbette et al., 2004; Cropley et al., 2006) demands during reading and may compensate for decreased connectivity within cortical pathways in dyslexic children. For example, dyslexic, relative to TR, may exhibit increased functional connectivity between the right thalamus and other regions during a verbal working memory task (Wolf et al., 2010), and atypical thalamic activity during reading-related tasks (Rumsey et al., 1997; Brunswick et al., 1999; Georgiewa et al., 2002; Ingvar et al., 2002; Hoeft et al., 2007; Pugh et al., 2008; Diaz et al., 2012). Whereas dyslexics have been reported to show both hypoactivation and hyperactivation of the thalamus during reading-related tasks, a meta-analysis revealed a general tendency toward hyperactivation of the right thalamus (Maisog et al., 2008) and other fronto-striatal circuits (Hancock et al., 2017) in dyslexic readers. Although the presently observed increased FA of the bilateral ThR would be in line with these findings, to further understand the functional significance of the structural differences in the ThR, it would again be essential to follow thalamo-cortical tracts during dyslexic and TR development using a longitudinal and/or intervention study design (e.g., Keller and Just, 2009) and to combine structural and functional data.

Furthermore, in line with verbal working memory difficulties, the dyslexic group in our study showed worse digit span scores (e.g., Smith-Spark et al., 2003; Jeffries and Everatt, 2004; Swanson et al., 2009). Verbal short-term memory problems, as expressed in poor digit span, are typical for children with dyslexia (Galaburda et al., 2006; Peterson and Pennington, 2015; Majerus and Cowan, 2016). However, this poor verbal short-term memory in people with dyslexia is usually seen as a manifestation of their phonological processing deficit (e.g., Tijms, 2004; Galaburda et al., 2006; but see, e.g., Smith-Spark and Fisk, 2007; Menghini et al., 2011), and dyslexic children in our study exhibited expected phonological processing difficulties (phoneme deletion). Concordantly, we found a significant correlation between phoneme deletion RT and digit span (raw scores: *r* = −437, *p* = 0.033, standard scores: *r* = −0.401, *p* = 0.052). Additionally, across group correlation between FA and word reading accuracy did not depend on verbal working memory, i.e., digit span (controlling for age and group: nodes 54–61, *r_avg_* = 0.608, *p_fdr_avg_* = 0.001, controlling for age, group and digit span: nodes 54–60, *r_avg_* = 0.614, *p_fdr_avg_* = 0.002) nor was digit span a significant covariate in the analysis of covariance.

### Atypical FA Values Within Bilateral Dorsal Reading Pathways in Dyslexic Children

Our results indicate differences between TR and dyslexic children in dorsal (AF, SLF) language/reading pathways (Vandermosten et al., 2012b; Cummine et al., 2013; Dick et al., 2013). First, dyslexic children, as compared to typical readers, exhibited differences in FA values along the bilateral AF. Although a follow up analysis along 100 individual nodes failed to reach cluster-size corrected significance levels, it indicated a tendency for higher FA values in dyslexic children in the central portion of both left and right AF together with lower FA values in the frontal part bilaterally and the posterior (temporo-parietal) part of the left AF. Furthermore, our initial analysis indicated atypical FA values in the right SLF, but a follow up analysis failed to yield group differences along individual clusters of nodes. As a significant interaction in the initial ANOVA may be a result of the large number of nodes versus the relatively small number of subjects included in the model, and the *post hoc* analyses did not reveal significant group differences, results regarding the AF and SLF although consistent with the literature, should be taken with caution. The AF connects temporal lobe structures with the inferior frontal gyrus (Gullick and Booth, 2015). It is an important connection within the language network, connecting Wernicke’s and Broca’s areas in the left hemisphere (Catani et al., 2005), and their counterparts in the right hemisphere that have been associated with both language and visuospatial attention functions (Catani and Thiebaut de Schotten, 2008; Doricchi et al., 2008). The AF may represent a connection within the dorsal phonological route of the reading network (Vandermosten et al., 2012b). The AF can be divided into three segments, i.e., an anterior, long, and posterior segment (Catani et al., 2005), of which the long segment that connects Broca’s and Wernicke’s areas, seems to be particularly relevant for reading (Saygin et al., 2013; Vandermosten et al., 2015). The AFQ toolbox, which was used in this study, tracts direct connections between frontal and temporal regions of interest, corresponding to the long segment, but we cannot exclude the possibility that the fibers belonging to the other two segments were also included in the calculated tract profile, which could diminish the observed differences (Vandermosten et al., 2015). Lower FA values of white matter in the left temporo-parietal lobe of dyslexic readers, and specifically lower FA values of the AF tract, are well supported by empirical evidence (for excellent reviews see (Vandermosten et al., 2012b, 2016). In particular the importance of the left AF in the beginning stages of reading seems to be independent of the alphabetic/logographic nature of the script (Cui et al., 2016; Su et al., 2018), although its role may diminish over years in logographic scripts (Qiu et al., 2011). Paradoxically, in beginner readers of English, lower FA of the left AF (and ILF) may be associated with better reading performance (Yeatman et al., 2012a; Christodoulou et al., 2016, but see Langer et al., 2015; Wang et al., 2016). This dynamic and idiosyncratic nature of white matter development may be influenced by different factors, including genetic predisposition for dyslexia (Marino et al., 2014) and possible gender differences in the reading network (Huestegge et al., 2012; Evans et al., 2014).

Our results further point toward differences in the right dorsal reading pathways. Empirical support for the right AF as part of the reading network is not as abundant. However, an increasing number of studies indicated the importance of the right AF/SLF in verbal recall (Catani et al., 2007), spelling ability (Gebauer et al., 2012) and reading comprehension (Horowitz-Kraus et al., 2014). Further evidence for the importance of the right hemisphere for reading in the typical population comes from reported positive associations between reading scores and the right SLF, right anterior limb of the internal capsule and right IFOF (Lebel et al., 2013), and an increased rightward asymmetry of the SLF, together with a decreased leftward asymmetry of the IFOF in dyslexic children (Zhao et al., 2016). Importantly, right SLF (and AF) properties were found to be predictive of long-term reading gains in dyslexic children (Hoeft et al., 2011). Finally, both good and poor readers at familial risk of dyslexia may show a different maturation speed for the right SLF, with faster maturation in better readers (Wang et al., 2016). Thus, although large parts of the reading network are known to be primarily left lateralized, our findings extend previous evidence for an additional involvement of right hemispheric regions (Kinsbourne and Warrington, 1962; Taylor and Regard, 2003; Lindell, 2006) consistent with the notion that extreme left lateralization may not be beneficial (Sabisch et al., 2006; Catani et al., 2007).

A subset of the dyslexic children included in the current study participated in a previous fMRI study where they were found to exhibit deviant letter speech-sound integration in the left superior temporal cortex (Blau et al., 2010). Our current DTI results not only included an expected group difference in FA values along the left AF (e.g., Gullick and Booth, 2014), but differences extended to the right dorsal reading pathway as well as the bilateral thalamic projections. Together with the previous fMRI findings (Blau et al., 2010), these group differences may point toward a deficient or “prolonged multisensory engagement phase” in dyslexic children (Fan et al., 2014), and/or increased attentional (Crick, 1984; Cropley et al., 2006) or working memory (Crosson et al., 1999; Crottaz-Herbette et al., 2004; Cropley et al., 2006) demands during reading. Importantly, however, most of the FA values indicated group by age interactions, suggesting that some of these differences could be due to the age of our participants as different white matter tracts have different maturational time courses (Paus et al., 1999; Lebel et al., 2008; Johnson et al., 2014), and this maturation may also differ between TR and dyslexic children (Rollins et al., 2009; Yeatman et al., 2012a; Christodoulou et al., 2016). A number of DWI studies investigating reading and/or dyslexia in children indeed revealed the importance of age (Nagy et al., 2004; Qiu et al., 2011; Yeatman et al., 2012a; Krafnick et al., 2014; Wang et al., 2016; Vanderauwera et al., 2017). Thus, in order to disentangle specific effects of dynamic maturational changes and reading experience in explaining these different patterns of DWI results, it would be important to extend these findings in future longitudinal studies in children across different orthographies and reading levels.

Finally, although our results are in agreement with previous findings, both from Dutch (Vandermosten et al., 2012a; Vanderauwera et al., 2017) and English (e.g., Carter et al., 2009; Hoeft et al., 2011; Yeatman et al., 2012a; Fan et al., 2014) speaking children, we acknowledge possible limitations of the current study. First, due to the strict quality control for movement in our DTI data, and a restriction to children with 2 to 3 years of reading instruction, our final sample was relatively small, which makes it important to replicate our findings in future studies (Ramus et al., 2018). Another potential limitation is that half way through the experiment the acquisition protocol was adjusted due to a scanner update. Although we kept the protocols as comparable as possible (200 ms difference in repetition time and 1 ms difference in the echo time), and acquisition protocol was included as covariate in the analysis, we acknowledge that this may have lowered the power to detect white matter differences. Lastly, as the protocols used in this study were designed for classical diffusion tensor analysis, more advanced acquisition schemes, e.g., high angular resolution diffusion imaging (HARDI; Tuch et al., 2002) and reconstruction algorithms could be used in future studies for better identification of local crossings.

## Conclusion

The results of the current study indicate bilateral differences between dyslexic and TR children after 2–3 years of the reading instruction in a fairly transparent orthography. In particular, our results suggest higher FA values in the bilateral ThR in dyslexic children, together with atypical FA values in the bilateral arcuate and right SLF. Interestingly, FA values in the left ThR scaled with reading-related scores. Moreover, children’s age was an important additional predictor of FA values in these tracts, emphasizing the importance of considering dynamic maturational changes when interpreting group differences in structural connectivity between dyslexic and TR. Together, our findings suggest differential contributions of cortical and thalamo-cortical pathways to the developing reading network in dyslexic and TR.

## Author Contributions

GŽ, IT, GF, and MB analyzed the data. PG and LB designed the experiments. GŽ, IT, PG, GF, JT, MM, and MB wrote the article.

## Conflict of Interest Statement

The authors declare that the research was conducted in the absence of any commercial or financial relationships that could be construed as a potential conflict of interest.

## Footnotes

1 https://github.com/vistalab
